# Community Health Navigators for Cancer Screening Among Deaf, Deafblind, and Hard of Hearing Adults Who Use American Sign Language

**DOI:** 10.1007/s13187-024-02416-x

**Published:** 2024-02-27

**Authors:** E. Bergeron, R. Valdez, C. J. Moreland, R. Wang, T. Knight, P. Kushalnagar

**Affiliations:** 1 Center for Deaf Health Equity, Gallaudet University, Washington, DC, USA; 2 Department of Public Health Sciences and Department of Engineering Systems and Environment, University of Virginia, Charlottesville, VA, USA; 3 Department of Internal Medicine, Dell Medical School at the, University of Texas, Austin, TX, USA; 4 Department of Family Medicine, University of California at San Diego, San Diego, CA, USA; 5 Department of World Languages and Cultures, Sam Houston State University, Huntsville, TX, USA

**Keywords:** Deaf, Sign language, Hearing loss, Cancer prevention

## Abstract

Deaf, deafblind, and hard of hearing (DDBHH) individuals experience barriers to accessing cancer screening, including ineffective patient-physician communication when discussing screening recommendations. For other underserved communities, culturally and linguistically aligned community health navigators (CHNs) have been shown to improve cancer screening and care. A needs assessment study was conducted to identify barriers and gather recommendations for CHN training resources. A community-based participatory needs assessment was conducted from May 2022 to June 2022 using three focus groups. Eight were cancer survivors, six advocates/navigators, and three clinicians. All questions were semi-structured and covered screening barriers, observations or personal experiences, perceived usefulness of having a CHN to promote cancer screening adherence, and training resources that may be useful to American Sign Language (ASL)–proficient CHNs, who are also culturally and linguistically aligned. Out of 20 focus group participants, seven self-identified as persons of color. Data highlighted systemic, attitudinal, communication, and personal-level barriers as recurrent themes. The most frequently cited barrier was access to training that supports the role and competencies of CHNs, followed by cultural considerations, access to cancer guidelines in ASL, dialect diversity in sign language, and the health system itself. Unaddressed barriers can contribute to health disparities, such as lower preventive cancer screening rates amongst DDBHH individuals. The next step is to translate recommendations into actionable tasks for DDBHH CHN training programs. As a result, CHNs will be well-equipped to help DDBHH individuals navigate and overcome their unique barriers to cancer screening and healthcare access.

## Background

Deaf, deafblind, and hard of hearing (DDBHH) individuals who use American Sign Language (ASL) are a subpopulation of the disability community that experiences significant disparities related to cancer screening and to cancer-related health outcomes. Deaf men, who are eligible for age-appropriate cancer screening and have a family history of cancer, often face disparities in shared decision-making compared to hearing men. For example, in the use of prostate specific antigen (PSA), DDBHH men felt significantly less involved in shared decision-making with their healthcare providers compared to hearing men [1]. Studies have shown that individuals from other marginalized populations, who are also DDBHH, face additional health disparities. A recent study found a significant disparity in the diagnosis of hypertension among Black Americans, with DDBHH having lower rates of hypertension compared to hearing Black Americans, likely due to underdiagnosis [2]. Thus, there is a need not only to address the cancer-related health disparities experienced by the DDBHH community but also to do so in ways that account for intersectional experiences.

Communication barriers, such as the lack of access to sign language interpreters, contribute significantly to cancer health disparities among the DDBHH population, affecting cancer screening knowledge and access to cancer-related information. A study found that deaf smokers without interpreters at medical visits were less likely to be asked about lung cancer screening [3]. Communication barriers similarly affect cervical cancer screening rates among deaf women [4].

Cancer-health knowledge appears to be qualitatively lower among DDBHH individuals compared to their hearing counterparts, possibly due to the abundance of health information that is primarily text- or audio-based in the USA. Deaf young adults, compared to hearing adults, have lower knowledge of human papillomavirus infection (HPV) and vaccine effectiveness in preventing cervical cancer [5]. One way to reduce such a knowledge gap is to provide cancer information in ASL, which has been found to improve cancer knowledge among DDBHH individuals who use ASL [6, 7].

Compared to hearing adults, deaf young adults are also more likely to have limited knowledge of their own family medical history, which can prevent their healthcare providers from providing personalized counseling on their individual cancer risk [5]. This often stems from the difficulties that deaf individuals face with hearing family members who do not use ASL when it comes to participating in everyday family conversations and potentially important health discussions [5]. Literature has shown that patient involvement in decision-making leads to improved knowledge, increased adherence to recommended screening, and higher satisfaction levels [1, 4].

Community health navigators (CHNs) are healthcare professionals who play a crucial role in supporting patients and their families by helping them navigate the complex healthcare system and access the care they need. CHNs, who are already proficient in ASL and have lived experiences, will be the focus of our intervention testing. Language concordance and shared lived experiences are the primary reasons for projected success with navigating patients and their families through the healthcare system. This group can effectively address cultural and linguistic barriers, fostering trust between patients and the healthcare system, ultimately improving quality of care [8]. Although the effectiveness of CHNs in reducing health disparities has been evaluated with individuals from racially and ethnically underrepresented backgrounds [9-12], there is limited research on the effectiveness of culturally and ASL-proficient CHNs for DDBHH ASL users. Since DDBHH individuals face barriers to cancer screening that parallel those faced by other historically marginalized communities, a navigator-based intervention may also prove beneficial for this community. We conducted a needs assessment study to identify the (1) barriers faced by DDBHH adults in accessing preventive cancer screening and (2) training resources that may be useful to promote cancer screening adherence for DDBHH people.

## Methods

We conducted a needs assessment study from May 2022 to June 2022 using three focus groups (41% were people of color): eight DDBHH cancer survivors, six DDBHH advocates/navigators, and three DDBHH and hearing clinicians. We structured our interviews with a set of questions and had the opportunity to ask the following:

- With cancer survivors and their caregivers, we asked about their barriers in obtaining cancer screening, their observations of barriers that others may experience, and their own experiences of having or not having a CHN/patient navigator.
- With healthcare professionals and CHN/patient navigators, we asked a series of open-ended questions about issues such as their experiences working with DDBHH patients who required cancer screening and barriers observed, training resources that may be useful to CHNs, and helpful clinical simulation scenarios for CHNs to work with patients non-adherent to cancer screening.

All Zoom interviews were conducted in ASL, and voice interpreters were provided for meetings with healthcare professionals. The interviews were recorded for transcription purposes and were immediately destroyed after the transcription was completed by an ASL-fluent author. Member checking, a process for ensuring rigor in qualitative research in which transcripts are checked for accuracy by participants and feedback is incorporated into the final versions of the transcripts, was conducted with each focus group participant [13]. Two authors coded a total of six transcripts, while a third author coded only two, utilizing a deductive coding structure. The codes generated were then integrated into the discussion held by the two authors who coded a total of six transcripts. The findings were organized into parent codes (Appendix) based on the three domains of the study: barriers to cancer screening, CHN training needs, and accessibility.

## Results

### Barriers

#### Systemic Barriers

One recurrent systemic barrier mentioned regarding cancer screening is insurance. A cancer survivor shared her experience of not having insurance and finding a low-income clinic when she discovered a lump in her breast. The clinic ended up referring her for a mammogram and she had to fill out extensive paperwork to apply for financial assistance. With CHNs, DDBHH patients can better understand how health insurance works, especially in regard to payment and bills.

The second recurrent subtheme identified is lack of accessibility feature in patient portals for appointments. One participant expressed their frustration, stating, “When I type to book appointments through patient portals, there is no option/way to request interpreters.”

The third sub-theme recounted accessibility problems that occur when healthcare providers are not familiar with requesting interpreters, do not understand the importance of having interpreters, and/or have no previous experience working with DDBHH individuals. Some interpreters may lack medical proficiency or do not possess the appropriate level of ASL proficiency. These hurdles underscore the critical importance of scheduling interpreters with the right expertise. DDBHH CHNs serve as a cultural liaison, bridging the gap between DDBHH patients and healthcare providers, offering guidance and addressing barriers, while sign language interpreters facilitate communication between healthcare providers and DDBHH individuals. Both roles are crucial for ensuring accessible and effective healthcare for the DDBHH community, but they serve different functions.

A caregiver shared an example of the issue, “Some require that I go through the hospital first rather than speaking with the interpreting agency. And most of the time, hospitals do not know what I’m talking about.” This can result in leaving DBBHH individuals without appropriate communication accommodations or relying on video relay interpreting (VRI) services, which may not be suitable for all situations. One participant recounted a negative experience, stating, “the VRI platform kept disconnecting because of lousy signals and doctors were not looking at us directly…”.

#### Attitudinal Barriers

One of the most common barriers identified is the outside perception that DDBHH individuals are uneducated, leading interpreters and healthcare professionals to provide inadequate or condescending information. A cancer survivor expressed their frustration, expressing, “Doctors often are not sure if they need to explain things differently to us, or whether we would understand things. Doctors often freeze and think this way, ‘Do they understand what I just explained?” Another LGBTQIA-identifying cancer survivor emphasized the importance of having LGBTQIA-friendly doctors and interpreters at their ob/gyn appointments. The same participant also added that interpreters may voice their responses with an unconscious judgmental tone.

Another common attitudinal barrier is a lack of patience from clinicians, who may become frustrated when communication takes longer than usual. One cancer survivor shared a negative experience where clinicians looked down on them due to their lack of English proficiency and being Black. The survivor explained, “They looked down on me because I am Black. And because I was not English proficient. I do not write English very well, but I do understand some things. They did not have any patience when we wrote back and forth on a piece of paper, as they gave me an attitude.”

To reduce the impact of attitudinal barriers to patient-centered cancer care, CHNs will be trained to advocate for their DDBHH patients, ensuring that healthcare professionals and interpreters treat DDBHH patients with respect. They will undergo sensitivity training to understand unique challenges faced by intersectional identities.

#### Communication Barriers

One participant expressed that cancer-related information can be challenging to understand, as information with medical terminologies and complex concepts may not be accessible in ASL. A cancer survivor stated, “There were no interpreters. I had to write on a piece of paper with a pen back and forth. Sometimes I would need to write and ask what the “big words” meant. I was young at that time and had no idea what Pap smear was for and what the speculum tool was going to do. Most doctors often say this, ‘Oh, you’ll be fine. We’re just doing the pap smear.’ That information was not enough, as I wanted to know more.” DDBHH individuals lack access to incidental learning, which is the information and knowledge acquired through daily interactions and experiences. This can impact their comfort level with making appointments and participating in health-related discussions. A community health navigator who works with the DDBHH community shared their observation on the relationship between patients and their family members, “Because of the lack of communication, they often do not know their family history of cancer.” DDBHH individuals seeking cancer screening may rely on peers within the deaf community for guidance and important sources of information. A prostate cancer survivor recounted his experience of providing information to a DDBHH individual preparing to undergo the PSA test.

To effective reduce barriers to cancer communication for DDBHH individuals, CHNs will be trained on how to bridge the information gap by providing clear explanations of medical terminologies and concepts in ASL. They will be trained to encourage shared-decision making, allowing DDBBH individuals to ask questions, empowering them to participate in their healthcare decisions.

#### Language Barriers

A cancer survivor shared their personal experience highlighting the issue of misinformation caused by the assigned sign language interpreter. They recalled the incident, saying, “…the interpreter told me that I was not eligible for [name of therapy]. I asked them, “Why?” They explained the reasons. I never said those in the first place. The information was wrong because the interpreter had relayed the message incorrectly. How do we know if the message was relayed properly? We do not know.” Another participant, a breast cancer survivor who had relocated to the USA from another country, stressed the importance of taking the time to explain the meanings of medical jargon. She stated, “I have also let them know that I’m from [country] and cannot read any “large” words. I told them to take time to explain to me the meanings of any “large” words.”

To effectively reduce the impact of language barriers on low cancer health literacy, CHNs will learn the importance of cross-verifying information, verifying DDBHH patients’ messages directly, and ensuring clarity in communication, especially when dealing with medical terminologies. They will be trained to process the message from the interpreter, and then transmit in the mode most easily understood by the DDBHH patient.

#### Personal-Level Barriers

Embarrassment and stigma, prior trauma in healthcare, lack of accessibility to health care services, fear of finding cancer, and transportation were commonly shared barriers. A transgender participant bravely shared their personal experience regarding stigma, stating, “…I did not like revealing my body parts that I’m not comfortable with. I have kept it to myself since my early days of college – during my freshman year probably. It was more than eight or nine years of not taking the pap smear test. Finally, I decided to take the test after realizing. Working with interpreters and the system was challenging though. Some interpreters, I did not feel comfortable working with. This is because our community is small. And I wish there were more trans-friendlier interpreters. So, I could relate with them better…” Regarding the fear of diagnosing cancer, another transgender survivor shared an insight, expressing their observation about themselves and other members of the DDBHH community. They stated, “So yes, when they think of screening… they do think of cancer.”

To address personal-level barriers experienced by some DDBHH individuals, CHNs will be educated on the importance of creating a welcoming and inclusive healthcare environment for individuals with intersectional identities, while providing emotional support to alleviate the fear of finding cancer.

### Recommended Resources for CHN Training

After analyzing the frequency of comments, the recurring themes expressed by our focus group participants provided valuable insights into the recommended resource topics for CHN training. The following areas were mentioned, listed from most to least often mentioned: role and competencies, cultural considerations, cancer guidelines, language diversity, and the health system. In terms of resource types for CHN training, the participants identified the following categories: CHN communication, print, website, and training course.

#### Role and Competencies

Cancer survivors who understand the role and competencies of CHNs recognize their utility during medical visits. One cancer survivor shared their experience, “I remember there was a confusion between the doctor and the interpreter. There was a lot of repetition… For large words, I had to clarify more than once. With repetition, I could tell the interpreter became frustrated. If I had a CHN, I would have received more support in regard to emotion, awareness, and information. The interpreter is only there to translate, and that is it.” Another survivor expressed similar sentiment about the value of having a CHN, saying, “They could have given me tips and advice on how to prepare, the predictability, side effects and many different scenarios. That would relieve all of my questions and I would become more “ready” to move forward.” Illustrating the importance of patient education and support, a community health navigator shared an experience involving a DDBHH patient, “…I have the ability as a CHN to go inside the building and let them know that this person is anxious and needs to see what machines look like such as the cat scan and mammogram machine. They often do not understand the ASL sign for mammogram (signs *breast push – breast flat* in ASL). When they see the machine and videos, they often have a better understanding of what to expect. Education is a key to getting screening. Education is the key.”

#### Cancer Guidelines

A cancer survivor expressed the need for clearly explained and accessible cancer screening guidelines in ASL, “…CHNs can research and then sign in ASL and write a list in English. That’s a good one. Um, I would like to add something. I would like the CHN to have all questions listed ready. For example, for screening and after diagnosis, specific questions of what to ask can be listed – which stage, which grade, which treatment, are there any other options.”

#### Misinformation Due to Language Differences

A prostate cancer survivor shared, “Most common confusion among our deaf community members is thinking that the prostate itself are testicles. I keep telling them, “No, it’s inside the bladder. It is the driver of both the bladder and the sperm. We call them the driver.” They often say, “Oh, I didn’t realize.” Many deaf people have thought of this way, “Oh, does that mean your testicles have been cut off?” I told them, “No!” It’s interesting how they can understand the meaning differently.”

When cancer information is delivered visually in sign language, it is highly critical that the terminology is relayed conceptually accurate to maximize health literacy in the DDBHH individual who receives this information. Language code-switching was mentioned by a focus group participant as a recommended solution to prevent cancer misinformation. “Some people may have a different communication method—ASL, PSE, or SEE.” — a focus group participant, with another one stating that. “CHNs would need to be prepared to code-switch different communication styles.”

## Discussion

A table of class content solutions and resources.

**Table:** 

Observed barriers	Resources to be added to the training program for CHNs
Role and competencies	Visual tools for cancer detection — informative posters and/or visually engaging PowerPoints Delivery of information skills Effective communication techniques, including plain language and patient-centered communication Competency in converting medical jargons to ASL Advocacy training Health information and data searching skills Encourage the use of evidence-based practice and research Role-playing scenarios to enhance CHNs’ problem-solving and patient interaction skills Ensure communication is facilitated accurately between the healthcare provider, the interpreter and the DDBHH patient Empowerment tools Guidance on motivational interviewing techniques Resources on patient empowerment, self-management, and shared decision-making Emotional support Resource referrals A comprehensive list of resources, including deaf-friendly support groups, financial assistance programs, and community organizations
Cultural considerations	Cross-cultural trainings (trainings in deaf Black health) LGBTQIA + friendly care Education on gender identities, sexual orientations, and the use of inclusive language CHNs that represent ethnoracial groups Cultural competency, sensitivity, and humility training Unique healthcare barriers faced by DDBBH individuals
Access to cancer guidelines in ASL	In-depth explanation of screening procedures in ASL Education on early detection Risk factors and symptoms Education on cancer basics (i.e., types of cancer, cancer stages List of questions to ask doctor This includes role-play scenarios where CHNs guide DDBHH individuals in asking these questions Side effects from medications
Delivery of cancer information to prevent misinformation	Anatomy education Signs that explain the anatomy and/or encourage hands-on learning with anatomical models Sign language of other countries Provide referrals to translators Different communication methods — ASL, PSE, or SEE
Health system	Navigating insurance Different types of health insurance (i.e., private insurance, Medicaid, Medicare) Complex insurance terms and processes Scheduling medical appointments Strategies for using online scheduling systems or via video phone Interpreter services Guidelines for working effectively with interpreters Step-by-step guidelines for CHNs to follow when requesting an interpreter Accessibility requirements Legal requirements related to accessibility under the Americans with Disabilities Act (ADA)

Previous studies have shown that CHNs with lived experiences and similar identities can increase cancer screening adherence among individuals from historically marginalized racial groups. It is possible these findings can be extended to the DDBHH population that uses ASL. This study used focus group methodology to explore DDBHH individuals’ barriers to cancer screening and CHN training needs to promote screening adherence. Recurring themes included systemic, attitudinal, communication, and personal-level barriers. The systemic barriers arose from difficulties navigating a complex healthcare system (e.g., misunderstanding insurance companies’ role, patient portal limitations). Attitudinal barriers encompassed the biases individuals face due to DDBHH status as well as the intersectionality with their other identities. Communication barriers included barrier DDBHH individuals experienced in exchanging medical information within their own families and with healthcare professionals. Finally, there are challenges unique to individual circumstances or experiences that may impact an individual’s healthcare decision. Together, these factors, when unaddressed, contribute to healthcare disparities, specifically lower cancer screening rates among DDBHH individuals. Few clinicians are trained to care for DDBHH patients.

Our primary goal is to cultivate a CHN workforce for DDBHH members, who are also community members with lived experiences, culturally competent, and ASL proficient. This involves implementing a targeted training program that emphasizes cultural competence and the nuanced understanding of barriers faced by this community. By addressing these aspects in the CHN training, we aim to empower DDBHH CHNs, to serve as a powerful resource in enhancing cancer screening rates and supporting DDBHH patients as they navigate the complexities of the healthcare system.

## Data Availability

Data is not available for public use due to small sample size and need to protect participants' identities.

## Appendix. Parent and child codes

**Table:**

Systematic or programmatic barrier:	Insurance Patient portals Transportation
Attitudinal barrier:	Interpreters Clinicians
Communication barrier:	Information accessibility Patient-physician communication Sign language interpreter access
Importance of CHN resources:	CHNresource communication CHNresource_print CHNresource_wesbite CHNresource_course
CHN training resource:	Language diversity Deafblind Health system Cancer guidelines Cultural Role and competencies
Language barrier:	Protacticle communication/interpreting International sign language
Personal-level barrier:	Embarrassment/stigma Fear of finding cancer Prior trauma associated with lack of accessibility to healthcare services Prior trauma in healthcare
Accessibility — device:	Phone Tablet Laptop Computer MiFi
Accessibility — video:	Background Narrator
Accessibility — caption:	Text color Text size Hide/unhide Transcript
